# Methods to increase reporting of childhood sexual abuse in surveys: the sensitivity and specificity of face-to-face interviews versus a sealed envelope method in Ugandan primary school children

**DOI:** 10.1186/s12914-016-0110-2

**Published:** 2017-02-23

**Authors:** Anna Louise Barr, Louise Knight, Ivan Franҫa-Junior, Elizabeth Allen, Dipak Naker, Karen M. Devries

**Affiliations:** 1 Department of Global Health and Development, London School of Hygiene and Tropical Medicine, 15-17 Tavistock Place, London, WC1H 9SH UK; 2 Department of Medicine, University of Cambridge, Cambridge, UK; 3 School of Public Health, University of São Paulo, State of São Paulo, Brazil; 4 Department of Medical Statistics, London School of Hygiene and Tropical Medicine, Keppel Street, London, WC1E 7HT UK; 5 Raising Voices, 16 Tufnell Drive, P.O. Box 6770, Kamwokya, Kampala Uganda

**Keywords:** Child sexual abuse, Violence, Methodology, Reporting, Disclosure, Uganda, Face-to-face interviews, Africa, Sexual violence, Confidential methods

## Abstract

**Background:**

Underreporting of childhood sexual abuse is a major barrier to obtaining reliable prevalence estimates. We tested the sensitivity and specificity of the face-to-face-interview (FTFI) method by comparing the number of disclosures of forced sex against a more confidential mode of data collection, the sealed-envelope method (SEM). We also report on characteristics of individuals associated with non-disclosure in FTFIs.

**Methods:**

Secondary analysis of data from a cross-sectional survey conducted in 2014, with *n* = 3843 children attending primary school in Luwero District, Uganda. Sensitivity and specificity were calculated, and mixed effects logistic regression models tested factors associated with disclosure in one or both modes.

**Results:**

In the FTFI, 1.1% (*n* = 42) of children reported ever experiencing forced sex, compared to 7.0% (*n* = 268) in the SEM. The FTFI method demonstrated low sensitivity (13.1%, 95%CI 9.3–17.7%) and high specificity (99.8%, 95%CI 99.6–99.9%) in detecting cases of forced sex, when compared to the SEM. Boys were less likely than girls to disclose in the FTFI, however there was no difference in prevalence by sex using the SEM (aOR = 0.91, 95%CI 0.7–1.2; *P* = 0.532). Disclosing experience of other forms of sexual violence was associated with experience of forced sex for both modes of disclosure.

**Conclusions:**

The SEM method was superior to FTFIs in identifying cases of forced sex amongst primary school children, particularly for boys. Reporting of other forms of sexual violence in FTFIs may indicate experience of forced sex. Future survey research, and efforts to estimate prevalence of sexual violence, should make use of more confidential disclosure methods to detect childhood sexual abuse.

## Background

Child sexual abuse (CSA) is a global issue, and is associated with lifelong health and psychological consequences [1–4]. CSA encompasses a range of acts, from verbal sexual comments and unwanted touching, to forced sex. A recent meta-analysis reported a global forced intercourse prevalence of 9% for girls and 3% for boys under 18 years [5]. Gold-standard methods for measuring exposure centre around asking participants to self-report experience of specific acts of violence, to avoid subjective classification of what constitutes abuse. However, concern about under-reporting remains—in many settings, CSA is highly stigmatized, and blame can be placed on the victim, rather than the perpetrator. Without adequate safeguards, disclosure can potentially put participants at risk of retaliation, social exclusion and without access to their basic needs, if the perpetrator is someone they are dependent upon [6].

Various tools have been designed to measure the self-reported prevalence of CSA, and other sensitive behaviours which can be subject to under-reporting. The face-to-face interview (FTFI) is the most commonly used method; it is suitable in low literacy settings and allows interviewers to prompt participants, provide clarification and ask further questions based on responses [3, 7–9]. The success of FTFIs is dependent on the interviewer’s ability to build rapport and gain the trust of the respondent [3]. The International Society for the Prevention of Child Abuse and Neglect Child Abuse Screening Tool-Child International (ICAST-CI) is one tool specifically developed to identify child victimization which is typically administered via face-to-face interview or as a self-completed questionnaire [10]. The assisted-self completion questionnaire (ASCQ) is another widely used tool. Interviewers read out questions and their corresponding answers, whilst participants independently mark their responses. ASCQs tend to be completed by groups of young people, in settings which can be difficult to ensure privacy e.g. crowded classrooms. Furthermore, some authors argue that participants require relatively high literacy rates in order to follow the interviewer and complete the ASCQ [11]. The public environment may also reduce the likelihood of participants asking clarifying questions.

For settings with low literacy more confidential methods of disclosure have been developed; however there is currently no standardized mode of measurement for population surveys. The WHO multi-country study on Violence Against Women employed a low-tech anonymous method to assess exposure to CSA in adult women across multi-country settings [12]. In response to a question about their experience of CSA, women marked pictorial representations of ‘yes’ and ‘no’ and placed the marked card in a sealed envelope or bag full of other cards. The method was developed to increase the likelihood of obtaining a more complete estimate of the prevalence of CSA in women by increasing the confidentiality of respondents’ answers [12].

A limited number of studies have been published specifically investigating the effect of mode on the disclosure of CSA amongst children and adolescents in low and middle income country (LMIC) settings [11, 13–17]. These typically found that methods which allow for anonymous disclosures are associated with higher prevalence estimates [6, 14, 15]. Despite this, the most common mode of data collection is via FTFIs [3, 7–9]. Little is known about the factors that are associated with disclosure in FTFIs versus anonymous data collection methods among children. If certain factors are associated with under-reporting of sexual abuse in FTFIs, understanding these can inform efforts to improve interview methods to obtain more accurate prevalence estimates.

The aim of this study was twofold. Firstly, to compare the number of students who disclose experience of forced sex in FTFIs to a more confidential method of data collection, the sealed envelope method (SEM). This method was designed to be used by primary school children in a LMIC setting where literacy is low [18]. We chose to measure the prevalence of forced sex, rather than CSA in general, as it is one of the more severe and stigmatised acts of CSA, and more likely to be subject to under-reporting when using a FTFI tool. Due to the stigma associated with forced sex we hypothesized that more students would disclose experience of forced sex using the more confidential SEM. Furthermore, we tested the ‘diagnostic’ accuracy (sensitivity and specificity) of the FTFI tool to identify children who had experienced forced sex against the SEM. The SEM was assumed to be the gold standard method, given its increased confidentiality.

Secondly, to explore factors associated with disclosure in the FTFI and the SEM; we compared students who chose to disclose using both the FTFI method and SEM to those who chose only to disclose in the SEM. We also explored factors associated with an affirmative response in the SEM, to determine what differentiates students who did not disclose forced sex in the FTFI from those who did not experience forced sex. We hypothesized apriori that male students and older students would be less likely to disclose in the FTFI versus the SEM as the perceived stigma would likely be higher in these two groups due to their growing awareness of societal norms and gender roles (e.g. men should be more sexually assertive) [15].

## Methods

Our cross-sectional study was nested within a large two-arm cluster randomized controlled trial that evaluated the Good School Toolkit. The trial protocol and child protection strategy are described elsewhere [19]. Briefly, the sample represents larger schools in Luwero District, Uganda, which contain 80% of all primary school students in the District. Forty-two schools were randomly selected from a list of 151 eligible primary schools in the District. All schools approached agreed to participate. In 2014, within each school, up to date class lists were obtained for Primary Five, Six and Seven. A simple random sample of up to 130 students was selected from the class lists in each school; 92% of individual students participated.

### Data collection via the face-to-face interview

Students’ experience of violence was measured using the International Society for the Prevention of Child Abuse and Neglect Child Abuse Screening Tool-Child International (ICAST-CI), [10] with some adaptations from the WHO Multi-Country Study on Women’s Health and Domestic Violence against Women [12]. Both have been used extensively in research in multiple country settings. All students were asked about their experience of sexual violence (Table 1), including whether they had been coerced or forced to have sex by a school staff member, persons beside school staff or their first partner. All questions were pilot tested with a sample of young people in Kampala and were used in a previous survey [8]. All FTFIs were conducted on the school premises by a trained interviewer, within sight of others but at a distance where they could not be overheard, to protect the confidentiality of interviewees.

**Table 1:** Definition of key variables

Variable	Items	Coding
Self-reported disability	Do you have difficulty seeing, even if wearing glasses? Do you have difficulty hearing, even if using a hearing aid? Do you have difficulty walking or climbing steps? Do you have difficulty remembering or concentrating? Do you have difficulty with self-care, such as washing all over or dressing? Using your usual language, do you have difficulty communicating, for example understanding, or being understood? Response options: No difficulty, Some difficulty, A lot of difficulty, Cannot do this at all.	Coded 1 if answered with ‘some difficulty’; 2 if answered with ‘a lot of difficulty’ or ‘cannot do’; 0 if answered no or n/a to all items.
Reported experience of other forms of sexual violence	Has a teacher or other adult that works at your school ever done any of the following things to you: Teased you or made sexual comments about your breasts, genitals, buttocks or other body parts? Touched your body in a sexual way or in a way that made you uncomfortable? By “sexual way” we mean touching you on your genitals, breasts or buttocks. Showed you pictures, magazines, or movies of people or children doing sexual things? Made you take your clothes off when it was not for a medical reason? Opened or took their own clothes off in front of you when they should not have done so? Kiss you when you didn’t want to be kissed? Make you touch their genitals, breasts or buttocks when you didn’t want to? Touch your genitals, breasts or buttocks when you didn’t want them to? Give you money/things to do sexual things? Involve you in making sexual pictures or videos? Threaten or pressure you to have sex or do sexual things with them? Has anyone besides a school staff member ever: Disturbed or bothered you by making sexual comments about you? Kissed you, when you did not want them to? Touched your genitals or breasts when you did not want them to, or in a way that made you uncomfortable? Threaten or pressure you to make you do something sexual with them? *(Does not include forced sex)*	Coded 1 if answered yes to any of the items; 0 if answered no or n/a to all items.
Any experience of forced sex	Has a teacher or other adult that works at your school ever done any of the following things to you: Actually make you have sex with them by threatening or pressuring you, or by making you afraid of what they might do? Make you have sex with them by physically forcing you (have sex with you)? Has anyone besides a school staff member ever: Make you have sex with them, because they threatened or pressured you? Had sex with you, by physically forcing you? Thinking about the person you had/have your first relationship with: Did you ever have sex with them when you did not want to, because you were afraid of what they might do? Did that partner ever force you to have sex with them when you did not want to?	Coded 1 if answered yes to any of the items; 0 if answered no or n/a to all items.
True Positive Respondents	Disclosed experience of forced sex in both the FTFI and the SEM	Coded 1 if answered yes to ‘Any experience of forced sex’ and yes to the SEM question; 0 if did not answer yes to both
False Positive Respondents	Disclosed experience of forced sex in the FTFI but not the SEM	Coded 1 if answered yes ‘to Any experience of forced sex’ and no to the SEM question; 0 if did not answer in this pattern
False Negative Respondents	Disclosed experience of forced sex in the SEM but not in the FTFI	Coded 1 if answered no to ‘Any experience of forced sex’ and yes to SEM question; 0 if did not answer in this pattern
True Negative Respondents	Did not disclose experience of forced sex in either method.	Coded 1 if answered no to ‘Any experience of forced sex’ and no to the SEM question; 0 if did not answer in this pattern

### Development of a more confidential method of disclosure – the sealed envelope method

We adapted aspects of the different methods that have been tested in LMICs in order to develop a simplified mode of confidential data collection for our sample of primary school children, the sealed envelope method (SEM). Due to low levels of literacy, pictures of faces were used to elicit a response from respondents about their experiences of forced sex, a method used in the WHO multi-country study on Violence Against Women [20]. Three sets of faces were piloted, asking children whether they had ever had malaria, which is common in the area. Students understood that the happy face was associated with never having had malaria, and the sad face with having had malaria. There was no real preference for the face type, therefore we chose to use caricatures of human faces as some children indicated that these faces conveyed more emotion.

Part way through the FTFI, the interviewer asked all children to mark a card depicting the sad and happy faces (boy or girl, depending on the sex of the child) in response to the question: “Have you ever been forced to have sex against your will?”. The child was asked to mark the happy face if this had never happened to them, or the sad face if this had ever happened to them, from any person. The child was instructed to mark the card and place it in a sealed envelope out of sight of the interviewer. At the conclusion of the interview and once the child was out of view, the sealed envelope was marked with the student’s identification number.

The presence of an interviewer provided opportunities for clarification and prompts, with the potential of increasing the response rate, as seen with the use of informal confidential voting interviews (a technique that incorporates an informal variant of the FTFI, to build rapport, with a self-administered questionnaire or a secret voting procedure for more sensitive questioning) [21, 22]. Children sealed envelopes themselves out of sight of the interviewer, reinforcing the confidentiality of the method [23].

### Forced sex variables

The variable ‘Any experience of forced sex’ was created from questions in the FTFI about experience of forced sex from any perpetrator (Table 1). ‘True positive’ respondents were those that disclosed experience of forced sex in both the FTFI and the SEM; these individuals were assumed to have experienced forced sex and to have been correctly identified by the FTFI. ‘False negative’ respondents disclosed experience of forced sex in the SEM only; these individuals were assumed to have experienced forced sex but were not identified by the FTFI method. ‘True negative’ respondents did not report experience of forced sex in either method; these individuals were assumed to have not experienced forced sex and therefore to have been classified correctly by the FTFI. It is unlikely that children chose to over-report experience of forced sex due to the associated stigma, therefore we assumed that an increase in the level of reporting reflected greater accuracy in the mode of data collection. For the SEM, answers were classified as errors when students had left the faces blank or otherwise did not answer definitively.

### Other measures

Other variables used in analysis are described in Table 1. A combined variable for ‘Reported experience of other forms of sexual violence’ was devised using questions related to students’ experiences of sexual violence from any perpetrator, but did not include experience of forced sex.

### Ethics

The study was approved by the London School of Hygiene and Tropical Medicine Ethics Committee (6183) and the Uganda National Council for Science and Technology (SS2520). Consistent with other school-based questionnaire studies involving sensitive issues in Uganda, headteachers and students provided consent for participation. Parents of students were informed of the study through letters sent home with students and by informal meetings held at participating schools and could opt children out. A staff member from the school administration and a representative from the Good Schools Study team explained the study and emphasized the voluntary nature of children’s participation. Prior to interviews, informed consent was sought from each child using a consent form containing a simple description of the study procedure. The consent form was read out to each child, with emphasis placed on their right to stop the interview at any time and to decline to answer questions. If children were unable to provide informed consent (e.g. they had a disability that prevented them from reading the consent form or hearing it read aloud, or they did not understand the study procedures described), they were automatically excluded from the study. Children were informed that if the interviewer believed their safety were at risk, they would be obligated to refer them to local child protection officers.

### Child protection

All respondents who reported sexual violence (as well as exposure to other forms of violence) in the FTFI were referred onwards to receive services in accordance with our child protection strategy, which linked vulnerable children with appropriate services [19]. A trained counsellor was also available to any child requesting counselling. In order to provide support to those children who chose not to disclose their experience of forced sex in the FTFI, without identifying them to others, all children were offered opportunities for further support through group counselling sessions. Each case of forced sex, regardless of mode of disclosure, was discussed on a case by case basis. We were aware that in less well-developed child protection systems, children referred to authorities may not experience optimal responses [24]. Therefore each case was handled with careful consideration of the child’s best interests through discussions between the study team and our child protection partners, and followed up by the study team to ensure child protection obligations were met.

### Analysis

All analyses were conducted using STATA 13.1 (Stata, College Station, TX, USA) [25]. The distribution of student demographic characteristics and self-reported experiences were calculated for the full sample, for those who disclosed forced sex in the FTFI and for those who had disclosed experience of forced sex in the SEM. We calculated the prevalence of different responses to FTFI and to the SEM, including errors.

For sensitivity and specificity analyses, to be conservative, all errors in the SEM were classified as negative answers. Despite the FTFI method being the most widespread method of data collection in this field, the SEM was assumed as the ‘gold standard’ of obtaining accurate disclosures of forced sex due to its increased level of confidentiality.

Sensitivity was defined as the proportion of true positives correctly identified by the FTFI, and specificity the proportion of true negatives correctly identified by the FTFI [26]. These, as well as positive and negative likelihood ratios, were calculated using standard formulae [27]. Positive Likelihood Ratios (LR+) can be interpreted as how many times more likely a positive answer in the FTFI will occur for respondents who have experienced forced sex compared to those who have not [28]. The further LR+ is above 1, the stronger the evidence for experience of forced sex. Likewise, the LR- indicates how much less likely a negative answer in the FTFI will occur for those who have experienced forced sex compared to those who have not [28]. The further the LR- is below 1, the stronger the evidence that the respondent has not experienced forced sex.

To test factors associated with different modes of disclosure, we fit mixed effects logistic regression models, accounting for clustering by school and controlling for interviewer random effects. The models were built using a theoretical approach which pre-specified factors that may be associated with disclosure of forced sex. Taking only those who had experienced forced sex, we compared those who disclosed in both the FTFI and SEM (true positive respondents), to those who disclosed only in the SEM (false negative respondents), to explore what factors are associated with disclosure in the FTFI. We also compared those who disclosed in the SEM but not the FTFI (false negative respondents) to those who did not report forced sex in either method (true negative respondents), to explore what factors are associated with experience of forced sex among those cases not identified by the standard FTFI method.

## Results

### Characteristics of respondents

Data from 3843 students were included in our analysis; their demographic characteristics are summarised in Table 2. Just over half of students were female (*n* = 2116, 55.1%), with 50.8% (*n* = 1951) of students aged 13 to 14 years. One fifth of students reported some form of difficulty, with 2.2% (*n* = 85) reporting a disability. Of those who reported forced sex in the FTFI (*n* = 42), the vast majority were female (*n* = 40, 95.2%) and two thirds had also reported experience of other forms of sexual violence (*n* = 28, 66.7%). Of those who reported forced sex in the SEM (*n* = 268), 60.8% were female (*n* = 163) and 20.9% (*n* = 56) of students reported experience of other forms of sexual violence.

**Table 2:** Student demographic characteristics and self-reported experiences

	All students		Students that reported forced sex in the FTFI^a^		Students that reported forced sex in the SEM^b^	
	*N* = 3843	%	*N* = 42	%	*N* = 268	%
Sex						
Female	2116	55.1	40	95.2	163	60.8
Age (years)						
<13	1331	34.7	12	28.6	91	34.1
13–14	1951	50.8	25	59.5	137	51.3
>14	558	14.5	5	11.9	39	14.6
Primary Level						
P5	1385	36.0	24	57.1	117	43.7
P6	1321	34.4	12	28.6	78	29.1
P7	1137	29.6	6	14.3	73	27.2
Self-reported disability ^c^						
No difficulties	2958	77.0	21	50.0	189	70.5
Some difficulties	800	20.8	19	45.2	69	25.8
Disability	85	2.2	2	4.8	10	3.7
Attends an intervention school	1973	51.3	21	50.0	161	60.1
Reported experience of other forms of sexual violence	239	6.2	28	66.7	56	20.9

^a^ *FTFI* face-to-face interview method

^b^ *SEM* sealed envelope method

^c^ Reported a degree of difficulty in seeing, hearing, walking, remembering or concentrating, communicating or with self-care

### Prevalence of forced sex by each mode

There was a seven-fold increase in the number of disclosures of self-reported experience of forced sex using the more confidential SEM (Table 3). A total of 1.2% (*n* = 46) of responses using the SEM were classified as errors. Although this is a small proportion of the overall sample, in comparison to the rare event of a student marking the sad face, this was relatively high. For the FTFI, there was no equivalent and immediate measure of respondent errors.

**Table 3:** Reported experience of forced sex by mode of disclosure

Answers by mode of disclosure	*N* = 3843	Percent
Face-to-face Interview (FTFI) answers:		
Any experience of forced sex	42	1.1
Sealed Envelope Method answers:		
Negative answer (happy face ticked)	3529	91.8
Affirmative answer (sad face ticked)	268	7.0
Total number of errors:	46	1.2
Left blank	8	0.2
Error: both faces ticked/marked	11	0.3
Error: sad face ticked, with a crossed out tick on the happy face	1	0.0
Error: happy face ticked, with a crossed out tick on the sad face	19	0.5
Error: happy face with NO written on it	7	0.2

### Sensitivity and specificity of the FTFI method

The FTFI method demonstrated low sensitivity (13.1%, 95%CI 9.3–17.7%), and high specificity (99.8%, 95%CI 99.6–99.9%) compared to the SEM, leading us to conclude that the FTFI method had a weak ability to identify those students who had been forced to have sex (Table 4). The LR+ was 66.7 (95%CI 29.9–149.0), indicating that a disclosure in the FTFI was strong evidence of experience of forced sex. The LR- was 0.87 (95%CI 0.83–0.91), and close to one, therefore a negative answer in the FTFI was not a strong determinant of the child having never been forced to have sex.

**Table 4:** Sensitivity and specificity of the FTFI method compared to the SEM method for identifying cases of forced sex

	SEM^b^					
FTFI^a^	Affirmative		Negative		Total	
Affirmative	35	(0.9%)	7	(0.2%)	42	(1.1%)
Negative	233	(6.1%)	3568	(92.8%)	3801	(98.9%)
Total	268	(7.0%)	3575	(93.0%)	3,843	
Sensitivity and Specificity Analysis:						
			95% CI			
Prevalence		7.0%	(6.2%, 7.8%)			
Sensitivity		13.1%	(9.3%, 17.7%)			
Specificity		99.8%	(99.6%, 99.9%)			
Positive Likelihood Ratio		66.7	(29.9, 149.0)			
Negative Likelihood Ratio		0.87	(0.83, 0.91)			

^a^ *FTFI* face-to-face interview method

^b^ *SEM* sealed envelope method, assumed ‘gold standard’ mode of data collection

### Who chose to disclose in the FTFI method?

All students that reported experience of forced sex in the FTFI and SEM (true positive respondents) were female (*n* = 35, 21.5%) (Table 5). Students who reported forced sex in both methods had nearly six times the odds of disclosing experience of other forms of sexual violence than those who disclosed in the SEM only (false negative respondents). These girls disclosed both acts of sexual violence that were not forced sex, and experience of forced sex in the FTFI.

**Table 5:** Comparison of those who disclosed in the FTFI and SEM (true positive repondents) to those who disclosed in the SEM (false negative respondents)

	*N* = 268	Percent	aOR	95% CI	*P*
Sex					
Male	0	0			
Female	35	21.5	n/a^t^		
Age (years)					
<13	11	12.1	1		
13–14	19	13.9	1.11	(0.38, 3.25)	0.854
>14	5	12.8	2.00	(0.34, 11.71)	0.439
Primary Level					
P5	20	17.1	1		
P6	10	12.8	0.48	(0.18, 1.31)	0.154
P7	5	6.9	0.24	(0.06, 0.94)	0.040
Self-reported disability					
No difficulties	19	10.1	1		
Some difficulties	14	20.3	2.33	(0.89, 6.05)	0.083
Disability	2	20.0	1.35	(0.20, 9.32)	0.761
Reported experience of other forms of sexual violence	23	41.1	5.59	(2.23, 14.04)	<0.001
Intervention school	17	10.6	0.53	(0.21, 1.34)	0.178

*aOR* adjusted odds ratio, *CI* confidence intervals

^t^ not available due to small cell size

*P* values derived from Wald tests

Each factor is adjusted for the other variables in the model and controlled for interviewer random effects and clustering by school

### What are the characteristics of those who chose not to disclose in FTFIs?

Comparison of differences between those that did not report experience of forced sex in the FTFI but did in the SEM (false negative respondents), versus those who did not report forced sex in either method (true negative respondents) revealed no significant associations with age or sex. False negative respondents had more than three times the odds of reporting experience of other forms of sexual violence in the FTFI compared to true negative respondents (Table 6).

**Table 6:** Comparison of those who disclosed in the SEM but not the FTFI (false negative respondents) to those who did not report forced sex in either method (true negative respondents)

	*N* = 3801	Percent	aOR	95% CI	*P*
Sex					
Male	105	6.1	1		
Female	128	6.2	0.91	(0.69, 1.21)	0.532
Age (years)					
<13	80	6.1	1		
13–14	118	6.1	0.98	(0.71, 1.36)	0.906
>14	34	6.2	0.92	(0.56, 1.50)	0.725
Primary Level					
P5	97	7.1	1		
P6	68	5.2	0.71	(0.50, 0.99)	0.044
P7	68	6.0	0.88	(0.60, 1.27)	0.482
Self-reported disability					
No difficulties	170	5.8	1		
Some difficulties	55	7.0	1.19	(0.85, 1.65)	0.307
Disability	8	9.6	1.22	(0.55, 2.69)	0.630
Reported experience of other forms of sexual violence	33	15.6	3.32	(2.15, 5.14)	<0.001
Intervention school	144	7.4	1.60	(0.99, 2.59)	0.056

*aOR* adjusted odds ratio, *CI* confidence intervals

*P* values derived from Wald tests

Each factor is adjusted for the other variables in the model and controlled for interviewer random effects and clustering by school

## Discussion

Students were seven times more likely to disclose experience of forced sex using the more confidential SEM method, versus the FTFI method. The FTFI method had low sensitivity to detect cases of forced sex—a negative answer in the FTFI was not a strong determinant of the child never being forced to have sex. In this sample, boys were just as likely to have experienced forced sex; however they were less willing to disclose in the FTFI. Those who chose not to disclose in the FTFI but did disclose in the SEM were over three times more likely to report experience of other forms of sexual violence than true negative respondents. Disclosure of other forms of sexual violence, other than forced sex, could be a potential indicator for hidden experiences of forced sex amongst those individuals who are not willing to disclose their more sensitive and stigmatized experiences in the FTFI.

To our knowledge, our study is one of the first to specifically investigate the characteristics of those who choose to disclose in the FTFI, and those who may go undetected by FTFI (that is, true positive and false negative respondents). We used a large, broadly representative sample of primary school children and tested both data collection methods on the same individuals, in order to test the consistency of respondents’ answers. Some other studies on the topic have randomly assigned participants to different reporting methods, but used relatively small sample sizes, leaving open the possibility of non-equivalence between groups [13, 15].

In comparison to other studies that tested mode of data collection on disclosure of CSA on school children in sub-Saharan Africa, our estimations of the prevalence of forced sex in this population were lower [7, 13, 15]. However, the children in our sample were also relatively young, and we would expect a lower prevalence in this age group versus older adolescents who have comprised most other samples. Stoltenbergh et al. suggest that studies that use large population-based randomized samples tend to yield lower prevalence estimates, and may be more accurate than higher prevalence estimates from unrepresentative samples [29]. Our sample however only included children who were attending school and therefore excluded vulnerable groups such as ill or street children who may have experienced higher rates of CSA.

Our findings are broadly similar to other studies examining the levels of CSA disclosure with more or less confidential methods. The WHO Multi-Country Study on Violence Against Women, reported that the more confidential method increased the reported prevalence of CSA experience in all but one site, compared with the FTFI [12]. Only two countries in the study linked the respondents’ answers in the FTFI and the confidential method to compare disclosures by each mode. Despite the increase in disclosure using the more confidential method, they found at the individual level that not all those who disclosed in the FTFI reported their experience in the confidential method (false positive respondents) and vice versa (false negative respondents). The extent to which this occurred is not reported, preventing comparison with our sensitivity and specificity results.

Plummer et al. who tested an ASCQ against a FTFI using two overlapping subsamples of rural adolescents in Tanzania, similarly found increased reporting of forced sex by participants when using the ASCQ; 0.2% of girls disclosed experience of forced sex in the FTFI, and 12.3% in the ASCQ [11].

Our study found no difference in the reporting of forced sex between male and female students when using the confidential SEM. Our results are supported by Lindstrom et al. who found that boys were less likely to report experience of coercion or rape at sexual initiation in the verbal response method compared with the non-verbal response method [15]. For boys, forced sex subverts the expected gender norms of male strength and sexual assertiveness [15]. If both the perpetrator and victim were male, the additional stigma of being labelled a homosexual, particularly in a country where homosexuality is illegal, could discourage disclosures in the FTFI. If the perpetrator was female, male students may be less willing to disclose in order not to appear weak [15]. This may account for why boys in our study chose only to disclose in the more confidential SEM.

Reported experience of other forms of sexual violence could be a potential indicator of forced sex based on our findings. Those who reported forced sex in the FTFI and SEM (true positive respondents) were almost six times more likely to report experience of other forms of sexual violence compared to those who reported forced sex in the SEM only (false negative respondents). The difference could be a reflection of the interviewers’ inability to build rapport and trust with false negative respondents and the willingness of true positive respondents’ to disclose sensitive experiences or to seek help. Likewise, false negative respondents were over three times more likely to report experience of other forms of sexual violence than those who disclosed no experience of forced sex (true negative respondents). Further evaluation is required to assess why these respondents were not willing to disclose their full experiences of CSA and how the context of specific acts may have affected their willingness to disclose e.g. carried out by different perpetrators.

As research on CSA must rely on self-report, comparative analyses between various modes of data collection can help to assess the ‘diagnostic’ capability of tools to identify individuals who have experienced CSA. We have demonstrated that a more confidential method of disclosure identified almost seven times more individuals who had experience forced sex than the more commonly used FTFI method. Such analyses can also help detect groups which may be underrepresented in studies using FTFIs, and inform the development of data collection modes which encourage disclosure amongst these select groups. Furthermore, these studies can assist researchers in identifying potential predictors of forced sex in FTFIs, such as experience of other forms of sexual violence, in order to inform child protection strategies which focus on identifying and preventing at risk individuals.

### Limitations

Whilst the SEM offers a greater level of confidentiality to respondents compared to the FTFI, it is still likely to be subject to under-reporting due to the sensitivity of CSA. Respondents’ understanding and definition of forced sex would no doubt influence the number of disclosures. Although questions were standardized and piloted, the interpretation of forced sex may be ambiguous to some respondents, depending on the “victim’s perception of the differences in size, strength (and) intelligence” between themselves and the perpetrator [1, 4]. The fact that males clearly respond in a physiological manner (e.g. erection and ejaculation) might influence their perception of forced sex as something they desired and instigated [30]. Many studies in Uganda, and other countries in Sub-Saharan Africa, have highlighted the common practice of coercive sex, where older men or peers provide girls with money and gifts in exchange for sex [31]. Girls in this situation may not view such transactions for sex as coerced or forced and therefore may not report it.

During the informed consent procedure, children were told that if they disclosed serious abuse, we would have to inform our child protection partners. This may have discouraged some students from disclosing, especially if they feared retaliation or were dependent on the perpetrator. Additionally, the setting of interviews, within the school, may have discouraged students from disclosing experience of abuse if their teachers were the perpetrators.

Our study evaluated each method based on the number of affirmative disclosures of forced sex; however this is only one component of a tools’ adequacy in collecting sensitive information. Further studies should evaluate which tool the respondent prefers and how comfortable they feel during data collection, whether a tool provides an opportunity for a respondent to seek support and how new technologies such as tablets, which can reduce data entry errors, can be best implemented in these settings.

## Conclusions

For research studies focused on CSA it is important that the data collection mode and participation in such studies does not cause discomfort or harm to the participants. There is a real necessity for reliable data on CSA, particularly in LMICs, however such research comes with great responsibility and must be subject to the highest methodological and ethical standards. There is a need to develop standardised, valid, reliable and ethical tools to measure CSA exposure in settings with low literacy rates in order to more accurately assess the prevalence of CSA experiences in these populations.

We have demonstrated that incorporating a confidential method of disclosure significantly increases reporting of forced sex, and that disclosure of other experiences of sexual violence besides forced sex may be an indicator for hidden experiences of forced sex. Importantly, this study highlights that in this setting, boys and girls are equally at risk of forced sex, but boys are less likely to reveal such experiences in FTFIs. National estimates that rely on FTFI studies may be seriously underestimating the true prevalence of forced sex and CSA.

## Abbreviations

ACSQ: Assisted self-completed questionnaire
CSA: Child sexual abuse
FTFI: Face-to-face interview
LMIC: Low and middle income country
LR-: Negative likelihood ratio
LR+: Positive likelihood ratio
SEM: Sealed envelope method
WHO: World Health Organization
